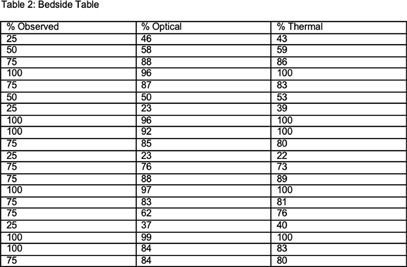# Use of an AI Platform for Monitoring Terminal Room Cleans

**DOI:** 10.1017/ash.2025.328

**Published:** 2025-09-24

**Authors:** Patricia Zuccaro, Palak Patel, Lizzy Urbina, Emily Landon

**Affiliations:** 1 The University of Chicago Medical Center; 2 University of Chicago; 3 UCMC

## Abstract

**Background:** The terminal cleaning procedure for disinfection of a hospital room is an essential, yet difficult to monitor, step in infection prevention. Current methods for auditing require intensive human resources investment, depend on self-report, or employ spot checks using ATPase or glo gel audits. Inconsistent feedback and self-report are notoriously poor ways to drive behavior change or evaluate quality. **Methods:** We assessed a new AI platform developed by Myna Technologies that monitors cleaning and disinfection. The system incorporates thermal and optical cameras to detect percentage of total surface area cleaned including adequate contact time for two high touch surfaces: bedside table and mattress, in our Environmental Services (EVS) training suite. A researcher, trained by our hospital’s EVS team, performed 20 cleaning passes each for the mattress and bedside table. Each pass covered 25%, 50% 75% or 100% of total surface area; we compared the planned cleaning percentage to the device-observed results for both cameras. A Fisher’s exact test analysis was performed for fully clean (100% surface) and not clean **Results:** See Tables 1 and 2 for cleaning plan and device results. The mattress was 100% cleaned 6 times of 20 passes. The thermal camera correctly identified complete clean 100% of the time **Discussion:** Initial assessment of this novel AI technology to monitor disinfection in real time shows promise for detecting adequate cleaning. A formal validation trial comparing results of the automated system with direct observation, glo-gel marking and ATPase for all high touch surfaces, utilizing multiple cleaners is underway.

**Figure:**
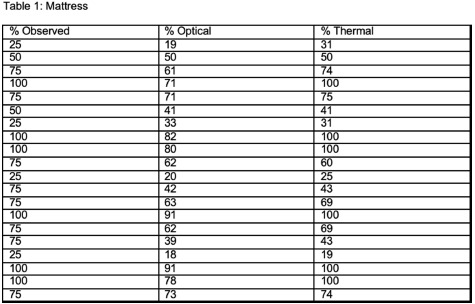

**Figure:**